# Higher CXCL16 exodomain is associated with aggressive ovarian cancer and promotes the disease by CXCR6 activation and MMP modulation

**DOI:** 10.1038/s41598-019-38766-6

**Published:** 2019-02-21

**Authors:** Hina Mir, Gurpreet Kaur, Neeraj Kapur, Sejong Bae, James W. Lillard, Shailesh Singh

**Affiliations:** 1 Department of Microbiology, Biochemistry and Immunology, Morehouse School of Medicine, Atlanta, GA USA; 2 Division of Preventive Medicine, UAB school of Medicine, Birmingham, AL USA

## Abstract

Ovarian cancer (OvCa) is the leading cause of death from gynecological malignancies. Five-year survival rate of OvCa ranges from 30–92%, depending on the spread of disease at diagnosis. Role of chemokines is well appreciated in cancer, including OvCa. However, their precise role is understudied. Here, we show clinical and biological significance of CXCR6-CXCL16 and ADAM10 in OvCa. Expression of CXCR6 and N-terminal CXCL16 was significantly higher in serous carcinoma tissues compared to endometrioid. OvCa cells (SKOV-3 and OVCAR-3) also showed higher expression of CXCR6 than normal ovarian epithelial cells (IOSE-7576) while CXCL16 was higher in SKOV-3 than IOSE-7576. Furthermore, N-terminal CXCL16 was higher in conditioned media of OvCa cells than IOSE-7576. Compared to OVCAR-3, SKOV-3 cells, which had higher CXCL16, expressed significantly higher transcripts of ADAM10, a protease that cleaves CXCL16. OVCAR-3 cells showed higher CXCR6 specific migration whereas SKOV-3 cells showed more invasion. Difference in invasive potential of these cells was due to modulation of different MMPs after CXCL16 stimulation. Higher CXCR6 expression in serous papillary carcinoma tissues suggests its association with aggressive OvCa. Increased migration-invasion towards CXCL16 implies its role in metastatic spread. Therefore, CXCR6-CXCL16 axis could be used to differentiate between aggressive versus non-aggressive disease and as a target for better prognosis.

## Introduction

OvCa is the fourth most common cause of cancer-related deaths in women. Its diagnosis and treatment still remain a challenge in gynecological cancer research. At present there are no means available to accurately screen women at risk of OvCa. Also the survival rate of OvCa patients is low even with combinatorial treatments due to intra-peritoneal metastasis. Identifying new mechanisms that play a role in OvCa progression will be of vital significance to facilitate not only timely detection of this disease but also design therapeutics aimed at decreasing metastatic risk.

Years of research have shown that a very controlled dysregulation of multiple biological pathways leads to development of cancer. Chemokine network is one of these dysregulated pathways. Under normal physiological conditions, chemokines and their corresponding GPCRs play an important role in the directional migration of hematopoietic cells and immune cells. However, cancer cells also exploit chemokine signaling for distant organ metastasis. Consequently, chemokines serve as key regulators of angiogenesis, cancer cell proliferation and metastasis^1–15^. Most studied of the chemokine signaling network is CXCR4-CXCL12 axis. This axis is important for bone marrow homing of hematopoietic stem cells, their quiescence and in neuronal guidance. However, this signaling axis is exploited during HIV infection as well as carcinogenesis. In OvCa, in particular, CXCR4 is overexpressed and correlates with reduced survival of patients. It is involved in promoting cell proliferation, invasion and metastasis. XCR1 is another chemokine receptor that is involved in promoting OvCa. Under normal conditions, it is expressed by dendritic cells and is important for dendritic cell mediated immune response, generation of Treg cells as well as induction of self-tolerance. However, activation of this receptor by XCL1 and XCL2 supports proliferation and migration of OvCa cells. Primary and metastatic OvCa cells also show increased expression of CX3CR1. This chemokine receptor plays an important role in neurons and microglial cell communication. However, it also significantly contributes to OvCa cell adhesion and proliferation. Owing to their diverse physiological roles, targeting these chemokine axes will be associated with neuronal and immune toxicity. Therefore, it is important to discover other mechanisms that could serve as more feasible therapeutic targets. Evidence suggests that CXCR6, following activation with CXCL16, plays an important role in leukocyte migration in atherosclerosis, rheumatoid arthritis, inflammatory diseases, and HIV infection. CXCL16 is expressed on immune cells, smooth muscle cells and endothelial cells. Like CX3CL1, CXCL16 can serve as an adhesion molecule in its membrane-tethered state as well as like chemo-attracting ligand in its cleaved, soluble state. Membrane bound form is anti-oncogenic whereas the soluble form functions in promoting cancer. Studies from our and other labs have revealed that CXCR6/CXCL16 axis is expressed in inflammation associated tumors, prostate cancer, breast cancer, lung cancer, renal cancer, colorectal cancer, pancreatic ductal carcinoma, nasopharyngeal carcinoma, and malignant melanoma^16–23^. Although, expression of CXCR6 and CXCL16 correlate with metastasis of OvCa to lymphnode and reduced patient survival the role of CXCL16/CXCR6 in OvCa is understudied. Interestingly, ADAM10, the protease responsible for cleaving CXCL16, is a proposed diagnostic and prognostic marker for NSCLC, laryngeal carcinoma and as marker for metastasis and poor prognosis of gastric cancer^24–26^.

In this study we have established the association of CXCR6 with aggressive phenotype of OVCa and have shown significance of CXCR6 and CXCL16 in the biological processes a cancer cell utilizes to establish metastatic lesions.

## Results

### CXCL16 and CXCR6 is highly expressed in OvCa tissues and cell lines

**Figure 1:**
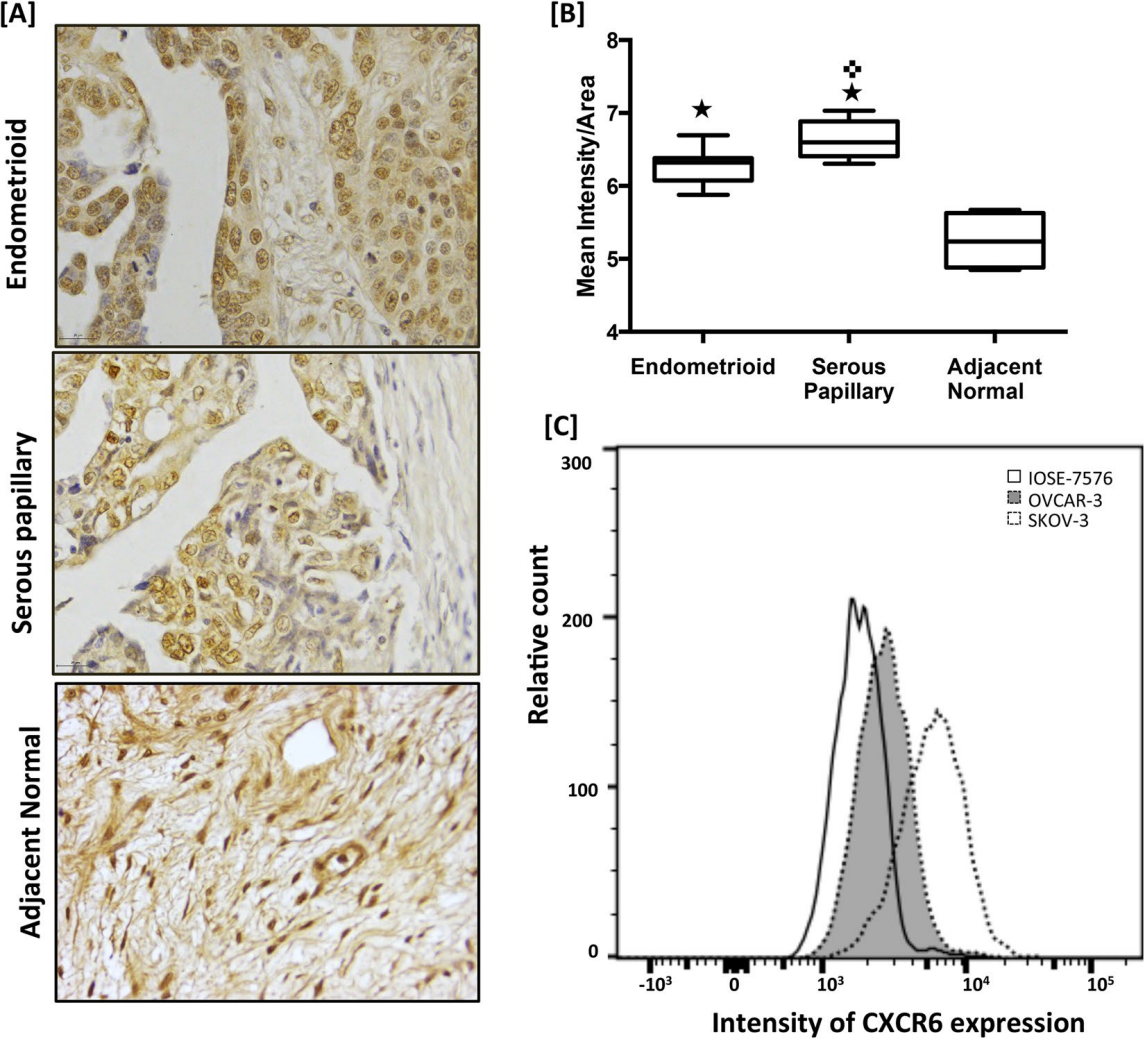
CXCR6 expression in ovarian cancer tissues and cell lines. Ovarian cancer tissues from normal (n = 12), endometrioid (n = 27) and serous (n = 33) were probed with antibody against CXCR6. CXCR6 was developed using DAB (brown). Images (40X) were captured using TissueFAXS cell analysis system from tissuegnostics (Panel A). Immuno-intensity of CXCR6 as shown in box plot was quantified using Histoquest image analysis software (Panel B). Line in the middle represents median value. ^★^p < 0.05 when compared to control; ^✜^p < 0.05 when compared between the endometrioid and serous papillary groups. CXCR6 protein expression in OvCa and normal epithelial cells was confirmed by FACS analysis. Cells were stained for CXCR6 expression using a purified phycoerythrin-conjugated anti-human CXCR6 (Panel C).

**Figure 2:**
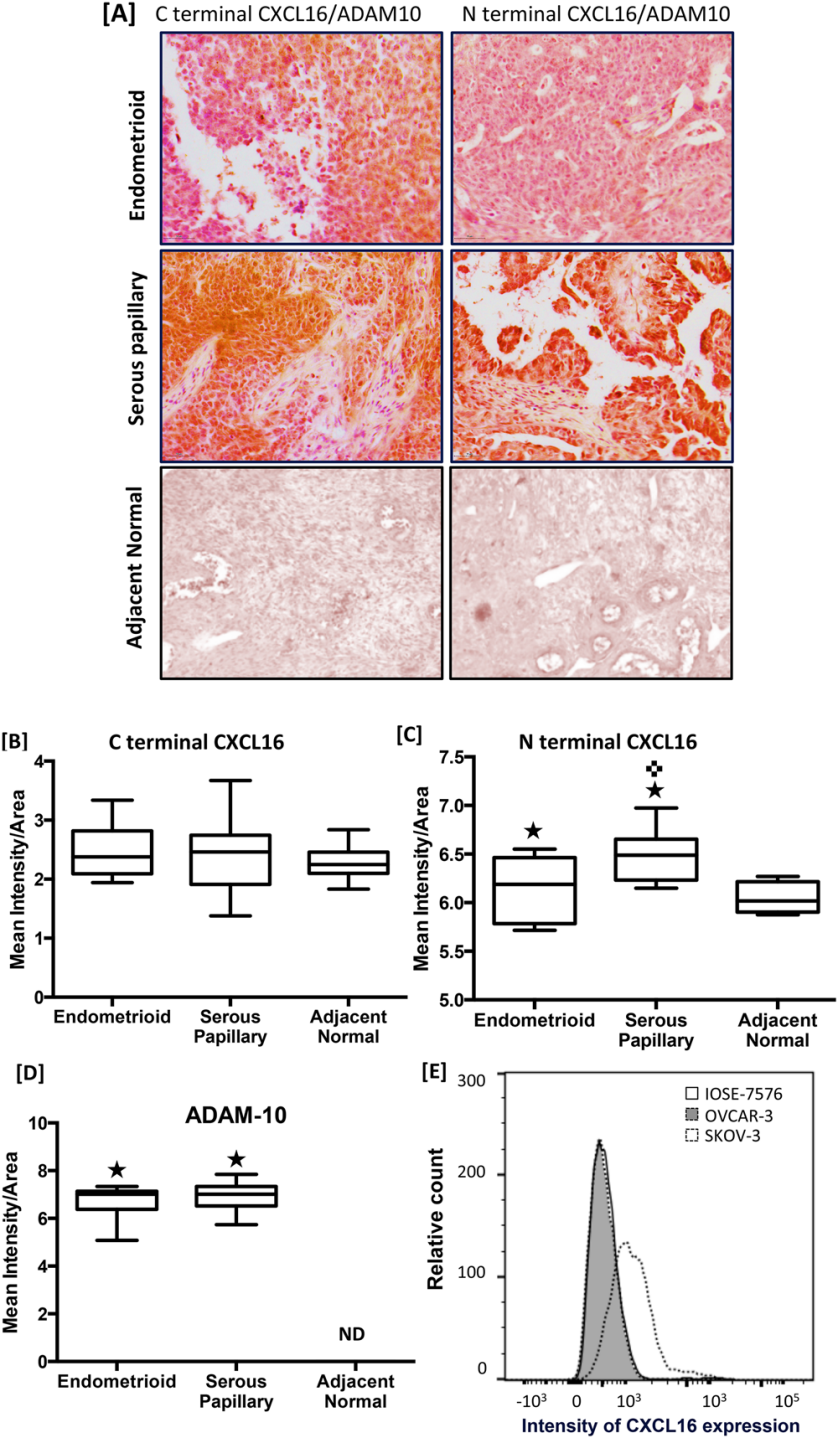
CXCL16 expression in ovarian cancer tissues and cell lines. Ovarian cancer tissues consisting normal (n = 12), endometrioid (n = 27) and serous (n = 33) were probed with antibody against N or C terminal of CXCL16 and anti-ADAM10 antibody. CXCL16 (N or C terminal) was developed using DAB (brown) and ADAM10 was developed using Fast red (Panel A). Images (20X) were captured using TissueFAXS cell analysis system from tissuegnostics. Immuno-intensities of C (**B**) and N terminal **(C)** CXCL16 and ADAM-10 **(D)** was quantified using Histoquest image analysis software and is shown in box plot. Line in the middle represents median value. ^★^p < 0.050 when compared to control; ^✜^p < 0.050 when compared between the endometrioid and serous papillary groups. No. ADAM-10 positive immunostaining (ND) was found in adjacent normal tissues. CXCL16 protein expression in OvCa and normal epithelial cells was quantified FACS analysis **(E)**. Cells were stained for CXCL16 surface expression using a purified APC-conjugated anti-human CXCL16 antibody.

OvCa TMA consisting of 33 papillary serous adenocarcinoma and 27 endometrioid adenocarcinoma tissues was stained for CXCR6 and CXCL16, the sole natural ligand of CXCR6. Immuno-intensity of CXCL16 and CXCR6 was quantified (Figs 1A,B and 2A–C). Expression of CXCR6 was significantly (p < 0.0350) higher in serous carcinoma tissues (Mean intensity/unit area = 6.6) compared to endometrioid tissues (Mean intensity/unit area = 6.323). Immune-intensity of N terminus CXCL16 was also significantly (p < 0.0409) higher in serous papillary carcinoma (Mean intensity/unit area = 6.488) compared to endometrioid (Mean intensity/unit area = 6.188) (Fig. 2A,C) whereas there was no significant difference in the immune-intensity of C terminus CXCL16 between serous papillary carcinoma and endometrioid (p = 0.1192) (Fig. 2B), however median intensity was higher in serous papillary carcinoma.

Expression of CXCR6 (Fig. 1C) and CXCL16 (Fig. 2E) was also quantified by flow cytometry in normal ovarian epithelial cells (IOSE-7576), highly invasive (SKOV-3) and less invasive (OVCAR-3) ovarian adenocarcinoma cell lines. Expression of CXCR6 was significantly higher in OvCa cell lines (SKOV-3 and OVCAR-3) compared to IOSE-7576. Expression of CXCR6 and CXCL16 was significantly higher in highly invasive OvCa cell line (SKOV-3) compared to less invasive OvCa cell (OVCAR-3) (Figs 1C and 2E). Cleaved CXCL16 level was significantly higher in conditioned media from OvCa cells compared to that from normal ovarian epithelial cells as quantified by ELISA. Further, CXCL16 levels in conditioned media of highly invasive SKOV-3 significantly (p < 0.05) higher compared to less invasive OVCAR-3, which confirms our FACS result (Fig. 2E).

### Ovarian cancer tissues and cells express ADAM 10

**Figure 3:**
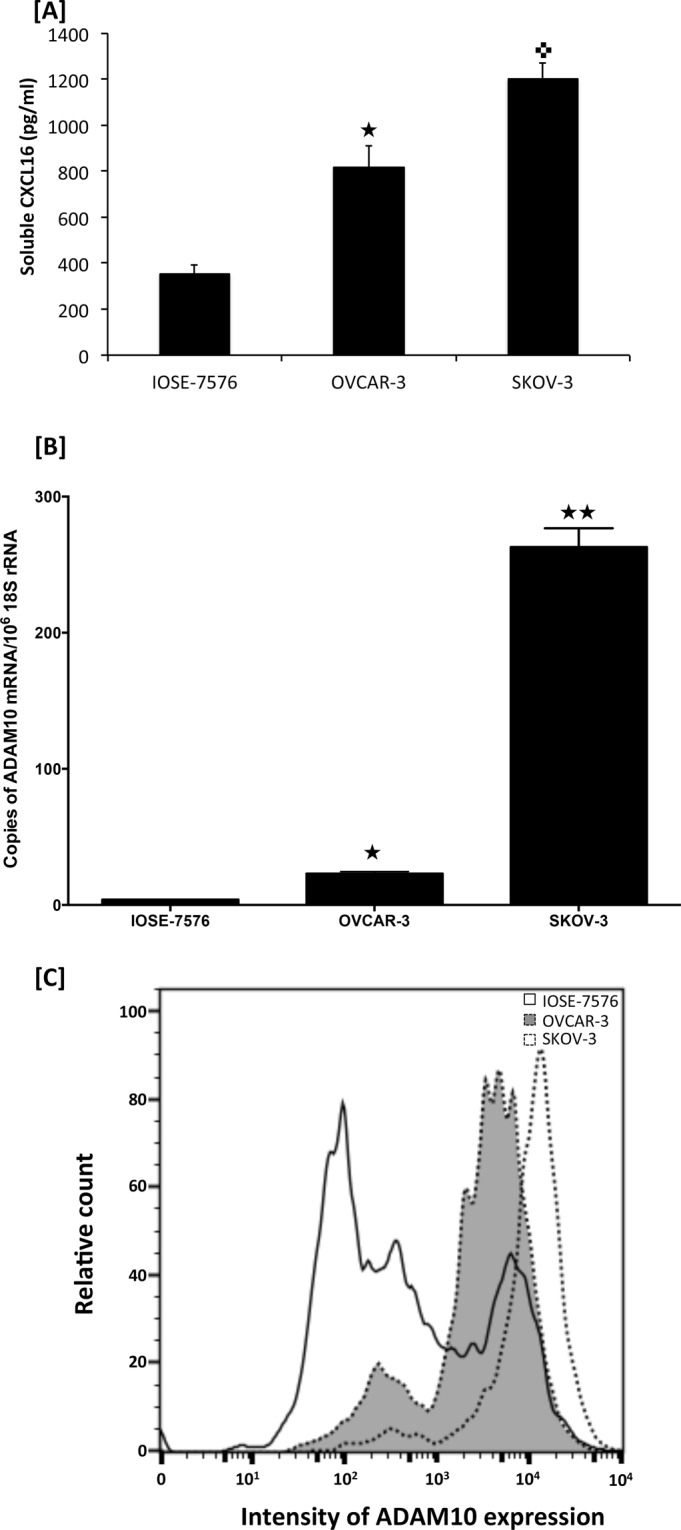
Levels of CXCL16 and ADAM10 in ovarian cancer cells. CXCL16 levels were measured in conditioned media of OvCa cells by ELISA. Values are mean ± SEM from 3 independent experiments (Panel A). ^★^p value < 0.05 when compared to IOSE-7576. ^✜^p value < 0.05 when compared to OVCAR-3. ADAM10 mRNA levels was quantified by RT-qPCR. Copies of ADAM10 transcripts are expressed relative to copies of 18 S rRNA. Values are mean ± SEM from 3 independent experiments (Panel B). ^★^p value < 0.05 and, ^★★^p value < 0.01 when compared to control. ADAM-10 protein expression in OvCa and normal epithelial cells was confirmed by FACS analysis. Cells were stained for ADAM-10 expression using a purified phycoerythrin-conjugated anti-human ADAM-10 (Panel C).

ADAM10, which cleaves membrane bound CXCL16 and activates CXCR6 was quantified in OvCa tissue and cell lines. Expression was detected in OvCa tissue but difference between histological types was not statistically significant (Fig. 2D). However, mRNA transcript of ADAM-10 in highly invasive SKOV-3 cells (262.9 ± 13.844) was higher compared to mRNA transcript (23.02 ± 1.566) in OVCAR-3 cells and the difference was highly significant (p < 0.05) (Fig. 3B). OvCa cells showed significantly higher ADAM-10 mRNA expression compared to IOSE-7576 cells (3.905 ± 0.12). Protein expression of ADAM-10 was confirmed by flow cytometry (Fig. 3C). Protein expression of ADAM-10 was same as we observed at mRNA level.

### Ovarian cancer cells migrate and invade in response to chemotactic gradient of CXCL16

**Figure 4:**
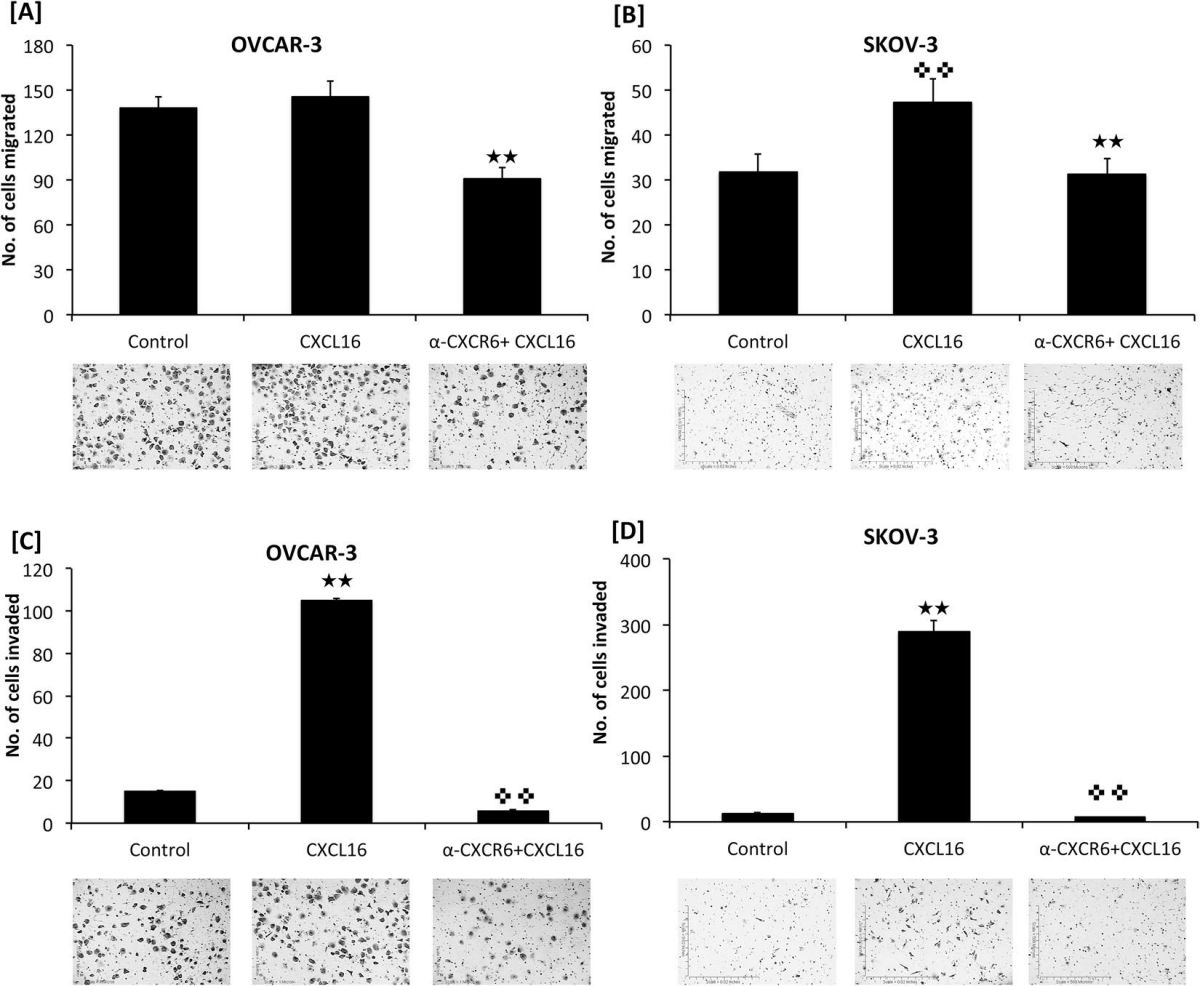
CXCR6-CXCL16 axis promotes ovarian cancer cell migration and invasion. Ovarian cancer cells (OVCAR-3 and SKOV-3) were allowed to migrate (Panel –A,B) or invade (Panel –C,D) in tumor migration and invasion chamber under the chemotactic gradient of CXCL16 (100 ng/ml) with or without anti-CXCR6 antibody (1.0 μg/ml) blocking of CXCR6. Cells that had migrated or invaded through the membrane or matrigel were fixed, stained and counted. Bars represent the mean ± standard error (n = 20) of number of migrated or invaded cells in response to CXCL16. ^★★^p < 0.01 represent statistically significant difference between cells migrating/invading towards CXCL16 gradient compared to control and ^✜✜^p < 0.01 represent statistically significant difference between cells migrating/invading towards CXCL16 gradient with and without CXCR6 blockade.

Biological significance of CXCR6 and CXCL16 was tested using tumor cell migration and invasion assays. OvCa cells migrated significantly towards CXCL16 chemotactic gradient. OVCAR-3 cells showed significantly (p < 0.0001) higher migratory potential (145.6 ± 10.57) compared to SKOV-3 cells (47.2 ± 5.35) in response to CXCL16. Migratory potential of OVCAR-3 and SKOV-3 in response of CXCL16 was significantly inhibited after CXCR6 blockade (p < 0.0001 and p < 0.0005, respectively) (Fig. 4A,B).

Matrigel invasion chamber consisting of Engelbreth-Holm-Swarm (EHS) mouse sarcoma containing laminin, collagen type IV, heparan sulfate proteoglycan, entactin was used to determine the invasive potential of OvCa cells under the chemotactic gradient of CXCL16. Both cell lines showed increased invasion under CXCL16 gradient compared to controls. In contrast to migration, SKOV-3 cells showed higher invasive potential compared to OVCAR-3 (p < 0.0001) (Fig. 4C,D), which was significantly inhibited (p < 0.0001) after CXCR6 blockade with anti-CXCR6 monoclonal antibody.

### CXCR6-CXCL16 axis differentially regulates expression of matrix metalloprotease (MMP) in OvCa cells

MMP play important role in digesting the matrix during invasion. Since OvCa cells showed different invasive potential in response to CXCL16, modulation in MMPs expression in response to CXCL16 was determined. Copies of collagenases (MMP-1, -8 and -13), gelatinases (MMP-2 and -9) and stromeylsins (MMP-3, -10 and -11) mRNA were quantified by RT-qPCR after 10, 30 and 60 minutes treatment with CXCL16. Levels of total MMP-1, -2, -3, -9 and -13 in conditioned media collected after 24 hours CXCL16 treatment were measured by ELISA. Active collagenases, gelatinases and stromeylsins were detected in the conditioned media 24 hours after treating with CXCL16 (Fig. 6D; Supplemental Fig. 1). Sample from untreated cells were used as control.

**Figure 5:**
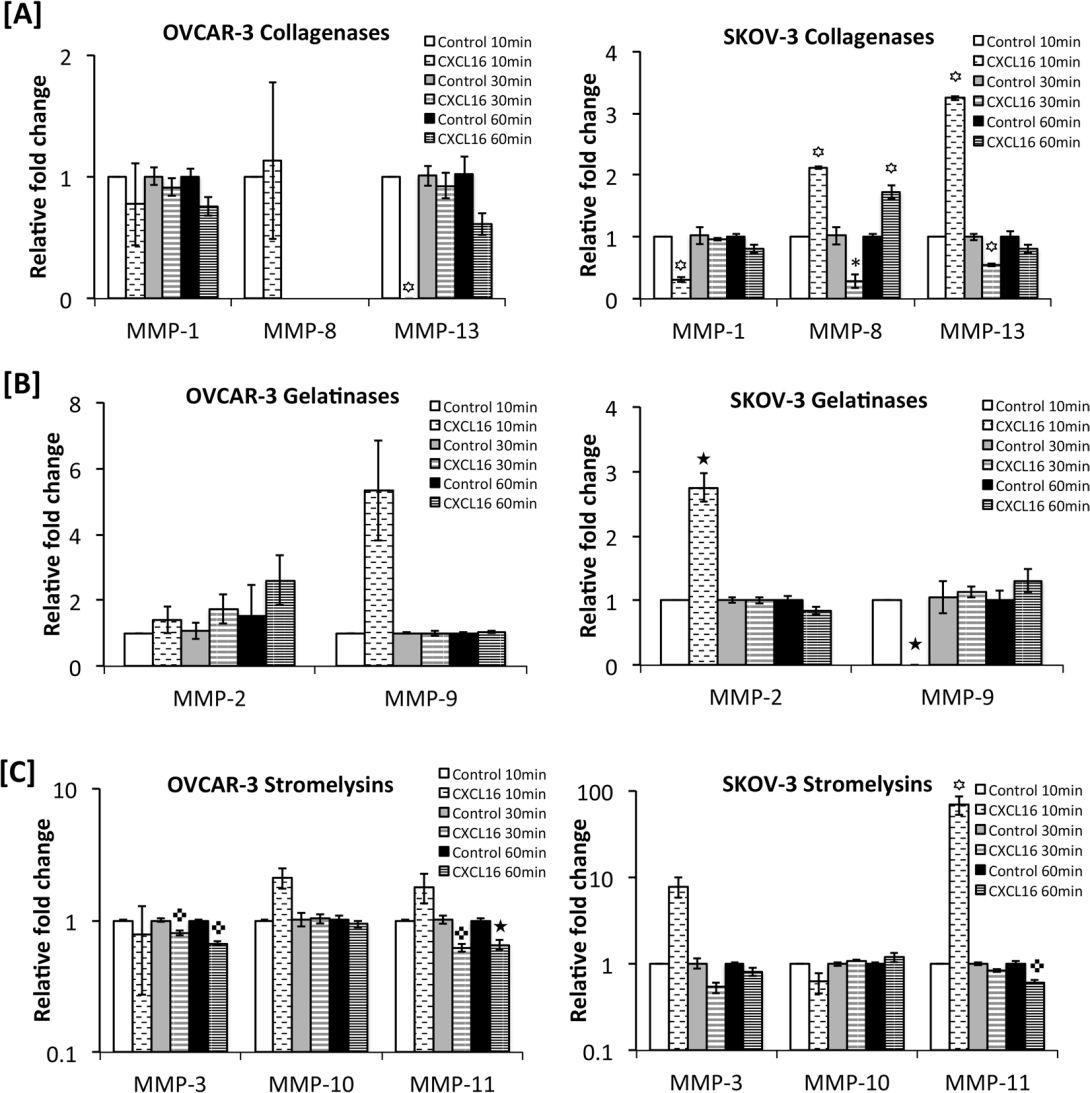
Expression of metalloproteinases (MMPs) transcripts by ovarian cancer cells in response to CXCL16 treatment. Changes in mRNA expression of Collagenases (MMP-1, -8 and -13) (Panel A), Gelatinases (MMP-2 and -9) (Panel B) and Stromelysins (MMP-3, -10 and -11) (Panel C) by ovarian cancer cell lines (SKOV-3 and OVCAR-3) after (100 ng/ml) CXCL16 treatment (10 min, 30 min and 60 min) were quantified by RT-qPCR. Relative quantities of MMP-specific PCR product were determined using the 2^−ΔΔCT^ method using 18S rRNA as internal control. Bars represent the mean ± SEM (n = 3). ^★^p < 0.05 and ^✜^p < 0.005 represent significant difference compared to respective untreated control.

**Figure 6:**
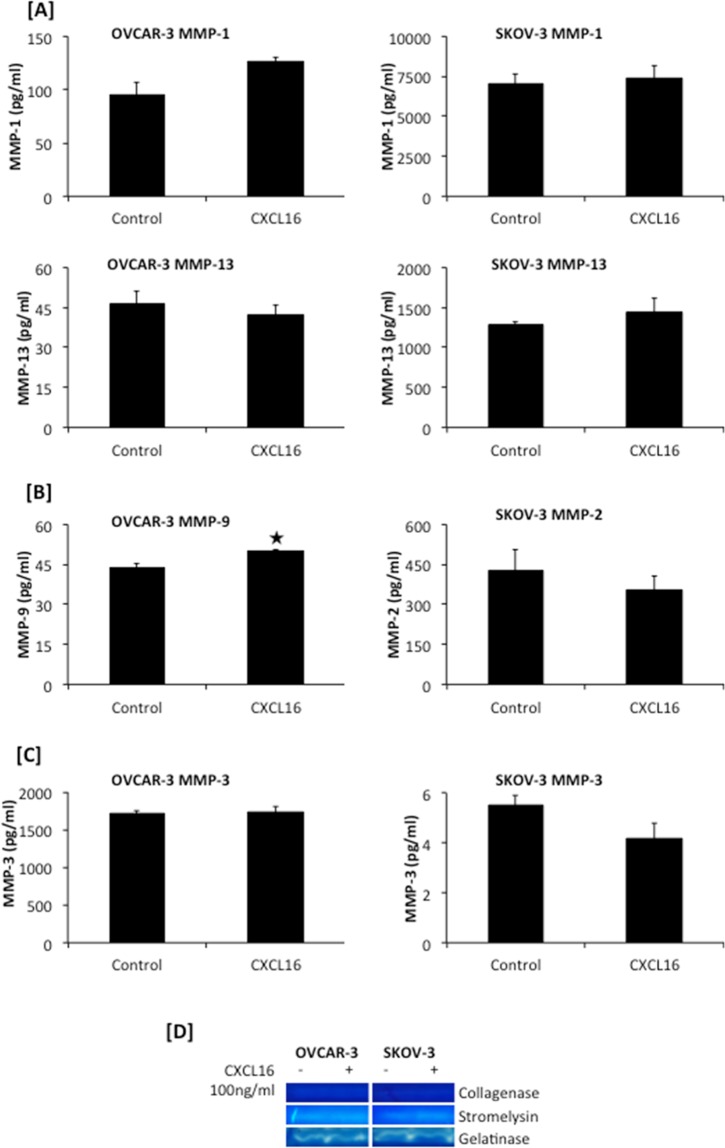
Levels of metalloproteinases (MMPs) in conditioned media of ovarian cancer cells treated with CXCL16. Changes in levels of Collagenases (MMP-1 and -13) (Panel A), Gelatinases (MMP-9 or -2) (Panel B) and Stromelysins (MMP-3) (Panel C) in the conditioned media of in ovarian cancer cells (SKOV-3 and OVCAR-3) treated with (100 ng/ml) CXCL16 for 24 hours were measured by ELISA. Bars represent the mean ± SEM (n = 3), ^★^p < 0.05 represents significant change as compared to control. Zymograms using casein, collagen and gelatin as substrate showing active stromelysin, collagenase and gelatinase in conditioned media of OvCa cells treated with and without CXCL16 (Panel D).

CXCL16 treatment significantly (p < 0.005) induced MMP-8 (10 and 60 minutes) and MMP-13 (10 minutes) mRNA expression in SKOV-3 cells. CXCR6 activation in SKOV-3 also led to significant reduction in mRNA transcripts of MMP-1 (p < 0.005) (10 minutes), MMP-8 (p < 0.05) (30 minutes) and (p < 0.005) MMP-13 (30 minutes) (Fig. 5A). Protein level of MMP-1 and -13 in the conditioned media of SKOV-3 cells treated with CXCL16 did not change significantly (Fig. 6A–C). OVCAR-3 did not show comparable changes in collagenases after CXCL16 treatment. CXCR6 activation in OVCAR-3 led to significant reduction in mRNA transcripts of MMP-13 (10 minutes) (Fig. 5A). Protein level of MMP-1 in the conditioned media of OVCAR-3 cells increased after CXCL16 addition however, this increase was not statistically significant. MMP-13 protein level also did not change significantly after CXCL16 treatment of OVCAR-3 cells (Fig. 6A). Collagenase activity could not be detected in OvCa cells (Fig. 6D).

Gelatinase- MMP-2 was significantly upregulated while and MMP-9 was significantly downregulated at mRNA levels in CXCL16 treated SKOV-3 cells after 10 minutes of treatment (p < 0.05) (Fig. 5B). However, MMP-2 protein levels did not change significantly after CXCL16 treatment. MMP-9 protein levels could not be detected as measured by ELISA in SKOV-3 cells (Fig. 6B). In OVCAR-3 cells, gelatinases mRNA transcripts did not change significantly after CXCL16 treatment (Fig. 5B). However, while MMP-2 could not be detected, there was a significant increase in MMP-9 protein in conditioned media of OVCAR-3 cells after CXCL16 treatment (Fig. 6B). Gelatinase activity also increased significantly after CXCL16 treatment (Fig. 6D).

Stromelysin, MMP-11, mRNA was significantly (p < 0.005) induced as early as 10 minutes while it was downregulated by 60 minutes of CXCL16 treatment of SKOV-3 cells (Fig. 5C). MMP-3 protein did not change significantly with CXCR6 stimulation and MMP-11 levels were below detection in the conditioned media of SKOV-3 cells (Fig. 6C). In OVCAR-3 cells, MMP-3 and MMP-11 mRNA were significantly (p < 0.005) downregulated 30 and 60 minutes after CXCL16 treatment (Fig. 5C). MMP-3 protein did not change significantly and MMP-11 levels were below detection in the conditioned media of OVCAR-3 cells (Fig. 6C). Stromelysin activity significantly higher in OvCa cells in response to CXCL16 treatment (Fig. 6D).

## Discussion

Late diagnosis of epithelial ovarian carcinoma leads to poor prognosis with the overall 5-year survival rate below 30%^27^. Patients with advanced-stage disease undergo cyto-reductive surgery and platinum-based chemotherapies, still relapse rate is over 70%^28^. Therefore, it is crucial to develop early stage biomarkers and define the molecular mechanisms of progression to develop betters therapeutics. In this study, we have emphasized the role of chemokine receptor CXCR6 and its ligand, CXCL16 along with that of ADAM-10 in supporting metastatic process of OvCa. CXCR6/CXCL16 axis is primarily expressed by immune cells and plays significant role in immune cell trafficking impacting innate and adaptive immune response against infection and cancer. Recent evidence from our group and others have established that chemokines are expressed in many cancer, including OvCa and exploit similar mechanism as used by the immune cells to survive, proliferate, migrate and invade to distant site^1–15,18^. We have shown clinical and biological significance of CXCR6-CXCL16 in lung and prostate cancer^16,18^. Recent studies have shown elevated CXCR6 and CXCL16 expression in OvCa patients with no significant difference between primary and metastatic tumor^29^. Guo *et al*. show a correlation between CXCR6 and CXCL16 expression with lymph node metastasis and poor survival^30^. In this study we have established the association of CXCR6 with different histological types. Higher expression in serous carcinoma that is aggressive histological type compared to endometrioid suggests association of CXCR6 with aggressive OvCa, which progress rapidly and poorly respond to therapeutic intervention. In addition to tissue expression our data also suggest higher expression of CXCR6 in SKOV-3 cells, which is more invasive and resistant to TNF-α, compared of OVCAR-3. Further, since CXCR6 is shown to be involved in stem cell self renewal and expressed by a subpopulation of melanoma cells^23^, higher expression of CXCR6 in OvCa may be involved in maintaining the stem cell-like phenotype contributing to higher relapse in OvCa.

CXCL16 is known to be involved in phagocytosis of bacteria by macrophages^31^ and also expressed by the lymphatic epithelium^32^ and inflamed tissues^33^. However, unlike other chemokines, CXCL16 also exists as a trans membrane protein, which has distinct biological function than soluble CXCL16 produced after CXCL16 cleavage by ADAM-10^34^. Our data show significant expression of CXCL16 in OvCa tissue. Although there was no significant difference in the C terminal CXCL16 levels between the two histological types of OvCa tissues, levels of N terminal CXCL16, was significantly higher in serous carcinoma compared to endometrioid. These observations correlated with increased level of cleaved CXCL16 in the conditioned media from OvCa cell line cultures. Cleaved CXCL16 (N-terminus) promotes proliferation^18^, survival and correlates with increased metastasis and poor prognosis^3,35^. Invasion assay provide evidence of similar role of cleaved CXCL16 in OvCa. Therefore, results showing significantly lower N-terminal CXCL16 in OvCa tissues of endometrioid type or high levels of soluble CXCL16, i.e. in conditioned media of OvCa cell lines strongly suggest significant contribution of this axis to the pathogenesis of OvCa patients and aggressive disease. Patients with serous papillary carcinoma with N-terminus CXCL16 higher than endometrioid appear to have some alternate mechanism like differential G- protein signaling after CXCR6 activation supporting their aggressive behavior.

ADAM10 has been associated with various cancers owing to its capacity to cleave exo-domain of CXCL16 that correlates with poor survival of OvCa patients. Level of ADAM10 was higher in endometrioid, which shows higher N-terminus CXCL16 but the difference in ADAM10 was not statistically different among different histological types. Levels of soluble CXCL16 in the conditioned media of SKOV-3 cells, which also express significantly higher ADAM10, was high compared to OVCAR-3. Together all these results suggest that ADAM10 and CXCL16 promote neoplastic progression of OvCa cells by working in tandem. However, obtained results also imply regulation of ADAM10 activity is more important than its expression as well as also raise a possibility of some alternate CXCL16 cleaving activity that may be determining the CXCL16 state. Also, of note is the fact that SKOV-3 cells are resistant to TNFα. TNFα leads to increased ADAM10 expression and hence release of CXCL16. This implies either TNFα independent mechanisms inducing ADAM10 activity operate in SKOV-3 or the cells are only partially resistant to TNFα, namely the signaling leading to apoptosis.

In addition to shedding CXCL16 from the cell surface ADAM10 could cleave E-cadherin and L1CAM thereby affecting the cell-cell adhesion. Soluble L1CAM thus generated promotes cancer cell migration via αVβ5 integrin in ERK and FAK dependent manner. We have also shown the role of CXCL16 in supporting cell migration via αVβ3 integrin in prostate cancer cells. ADAM10 is also reported to increase migration potential of non-small lung cancer cells through Notch 1 signaling pathway. These facts corroborate with the data showing increased migration of the two OvCa cells by CXCL16.

One of the most important steps in tumor progression and metastasis is the invasion of surrounding tissues by the neoplastic or malignant tumor cells. This requires chemotactic migration of cancer cells and attachment of their cell membrane to the extracellular matrix. CXCR6-CXCL16 axis promotes invasion of lung and prostate cancer^18,19,29^. In the current study we observed selective invasion of high CXCR6 expressing OvCa cells towards CXCL16 in a CXCR6-dependent manner. Therefore, this axis plays important role in promoting metastatic capacity of OvCa.

In order to modulate the extracellular matrix cancer cells invariably lease matrix metalloproteinase system, irrespective of the upstream signals that trigger metastasis. Consequently, MMPs contribute to the carcinogenic process at multiple stages; high levels of MMP correlate positively with stage, invasive and metastatic properties and ultimately poor prognosis. They serve as accomplice in bringing about structural alterations in the extracellular matrix to facilitate migration and homing of tumor cells and are up regulated in variety of human cancers. CXCR6-CXCL16 interaction triggers cytoskeleton reorganization along with induction of MMPs promoting to invade through the extra cellular matrix^16^. We focused on CXCR6-CXCL16 mediated changes in OvCa MMPs.

Collagenases (MMP-1, MMP-8 and MMP-13) support digestion of collagen type I, II and III. Expression patterns of these are associated with the stage and type of tumor^36–38^. MMP-1 cleaves PAR-1, which promotes proliferation, endothelial tube formation, migration and invasion of OvCa cells^39,40^. MMP-8 expression was correlated with OvCa grade, stage and prognosis^41^. Upregulation of MMP-8 under pro-inflammatory conditions enhances the invading capacity of OvCa^41^ while MMP-13 is a prognostic marker and it negatively correlates with survival^37^. Stimulation with CXCL16 led to increase in MMP-8 mRNA and MMP-13 levels in conditioned media in SKOV-3, which express more CXCR6, while OVCAR-3 cells did not show significant change in these collagenases after CXCL16 treatment. However, no collagenase activity was detected after 24 hours in OvCa cells with or without CXCL16 treatment.

Gelatinases (MMP-2 and MMP-9) have been implicated in the induction of the angiogenic switch in different tumor models^42,43^. These are involved in degradation of structural protein of extracellular matrix and basement membrane and hence positively associate with tumor progression^44–46^. High levels of MMP-2 and -9 have been found to correlate with migration, invasion and poor prognosis of OvCa^47^. Levels of these MMPs in urine could serve as diagnostic markers for OvCa^48^. In the present study, OVCAR-3 and SKOV-3 cell lines expressed MMP-2 and -9 mRNA. Importantly, CXCL16 treatment induced MMP-2 mRNA by SKOV-3 cells only. CXCL16 treatment induced an increase in MMP-9 levels in conditioned media of OVCAR-3, but not that of SKOV-3 cells. Thus, after CXCL16 treatment expression levels of these two gelatinases do not change significantly, nonetheless, there was a significant CXCR6-CXCL16 specific invasion through the membrane coated with collagen type IV and growth factors like TGFβ, which implies increased activity of MMP-2 or -9 or both. This was confirmed by CXCL16 induced increase in the active gelatinases in the conditioned media of OvCa cells.

Stromelysins (MMP-3, -10 and -11) that digest an extensive range of substrates are expressed by normal epithelial as well as cancer cells. MMP-3 expression correlates with MMP-7 and -9 activation in ovarian tumors^49^, while MMP-10 expression correlates with OvCa migration and chemo-resistance^50,51^. MMP-11 expression correlates with ovarian carcinoma malignancy. Only SKOV-3 cells showed an induction of MMP-11 mRNA after CXCL16. However, enhanced invasion as observed in response to CXCL16 gradient could be partially attributed to an increase in the activity of MMP-3 and MMP-10, which degrade collagen type IV and laminin. MMP-3 could also contribute to the increased invasive potential of these cells indirectly by activating gelatinases and MMP-13. Increase in the active gelatinases in the conditioned media of OvCa cells after CXCL16 further corroborates with this possibility. Thus, stimulation with CXCL16 supports the increased invasive capacity of OvCa cells by activating MMPs without altering their expression remarkably.

In summary, we showed high expression of CXCR6 and CXCL16 in OvCa tissues and cells. Increased CXCL16 promote metastatic capabilities of OvCa cells in CXCR6 specific manner. We also showed high ADAM10 expression in OvCa cells that would promote release of CXCL16 from the membrane. Finally, we showed CXCL16/CXCR6 interaction led to increased migration and invasion that could be due to activation of MMPs. Therefore, our findings indicate that ADAM10-CXCL16-CXCR6 axis could be used as the therapeutic target to inhibit OvCa metastasis. Also, serum CXCL16, representing soluble form of CXCL16, could be used as a non-invasive biomarker for OvCa detection and prognosis. This, in addition to our past findings, implies that CXCR6-CXCL16 axis is widely exploited by cancer cells to survive and progress in various malignancies. Hence, therapy directed to this axis will provide new option for cancer treatment.

## Methods

### Cell culture

Human OvCa cell lines (SKOV-3 and OVCAR-3) were obtained from the ATCC (Manassas, VA). Immortalized ovarian surface epithelium (IOSE-7576) cells were used as non-neoplastic control. OVCAR-3 cells were cultured in RPMI-1640 (ATCC, MN, USA), supplemented with 0.01 mg/ml bovine insulin, 20% fetal bovine serum (FBS, ATCC, Manassas, VA). SKOV-3 cells were cultured in McCoy’s 5a Medium supplemented with 10% FBS. IOSE-7576 cell were cultured in DMEM supplemented with 10% FBS. Penicillin (100 U/ml) and streptomycin (100 μg/ml) was added to media. The cells were maintained at 37 °C in a humidified atmosphere of 5% CO_2_.

### Tissue specimens

Tissue microarray (TMA) slides containing ovarian tissues and adjacent normal  were procured from Cooperative Human Tissue Network (CHTN), UAB, Birmingham and US Biomax, Inc. We have used retrospectively collected de-identified tissue in this study, which was conducted as per guidelines of IRB of Morehouse School of Medicine. TMA consisted of 33 papillary serous adenocarcinoma and 27 endometrioid adenocarcinoma tissue samples. A qualified pathologist confirmed the histopathology, the class and the grade of the tumor validated each core of the tissue microarray.

### Immunohistochemistry

TMAs were deparaffinized in xylene, rehydrated through a graded series of ethanol (100%, 95% and 70%) for 5 minutes in each and washed with distilled water. For antigen retrieval, TMAs were incubated with 0.01 M EDTA (pH 9.0) in a pressure cooker for 5 minutes and then cooled in running water. Slides were then transferred to Tris-buffer (pH 7.6) and the endogenous peroxidase activity was blocked by incubating with 3% H_2_O_2_ in PBS for 3 minutes. After rinsing with deionized water followed by 3 washes in Tris-buffer, sections were blocked with avidin for biotin for 10–15 minutes, washed with Tris-buffer and blocked for Biotin with biotin block for 10–15 minutes followed by 20 minutes incubation with 3% horse serum at RT. After blocking, TMA was washed with Tris-buffer, and incubated for 1 hour in humidity chamber at RT with mouse anti-human-CXCR6 monoclonal antibody (1:75 dilution of 1.0 μg/ml stock) (R&D Systems). Isotype was used as a negative control (R&D Systems). After washing, sections were incubated at RT for 20 minutes with multi-species link (BioGenex, CA). Next, sections were incubated with multi-species label (BioGenex, CA) for 20 minutes at RT after removing link, developed with DAB (BioGenex, CA) for 5 minutes and counter-stained with Mayer’s hematoxylin (Sigma) for 1 minute and washed with DI water. After de-hydration with alcohol and xylene slides were mounted with permount (Sigma).

CXCL16 and ADAM10 double staining were performed on serial sections from same TMA block. Dehydration, antigen retrieval and blocking were same as described above for CXCR6 staining. Sections were incubated at RT overnight with antibody (1:50 dilution from 1.0 μg/ml stock) against C-terminus of CXCL16 raised in goat. Additional TMA was used for staining N-terminus of CXCL16. Sections were incubated at 37 °C for 1 hour with 1:50 dilution of CXCL16 antibody raised in goat against N-terminus of CXCL16. After washing the primary antibody both TMAs were incubated at RT for 20 minutes with monoclonal anti-goat/sheep IgG–biotin antibody produced in mouse (Sigma). Next, sections were incubated with multi-species label (BioGenex, CA) for 20 minutes at RT after removing link and developed with DAB (BioGenex, CA) for 5 minutes. After developing for CXCL16 N- and C-terminus, both TMAs were blocked using goat affinity purified Fab fragment of goat anti-mouse IgG H + L 20 ug/ml (Jackson Immuno Lab) for 20 minutes at RT, sections were blocked with 3% house serum. Sections were incubated for 1 hour at RT with ADAM10 (1:50 dilution) antibody after washing horse serum. TMAs were incubated at RT with multispecies link. Link was washed with Tris buffer and incubated with alkaline phosphatase (AP)-conjugated goat anti-mouse antibody (Zymed) for 20 min at RT. Sections were then washed and incubated with the AP New magenta (BioFX Laboratories) for 25 minutes at RT. After washing the label sections were counter-stained with Mayer’s hematoxylin (Sigma) for 1 minute and washed with DI water. De-hydration was done in 70%, 95% and absolute alcohol for 5 minutes each and then the slides were passed through xylene thrice for 1 minutes each and mounted with permount (Sigma).

### Immunohistochemical evaluation of CXCR6/CXCL16 and ADAM10

TMA were scanned using Tissuegnostics microscope using TissueFAXS software. Images were exported to analysis (Histoquest) software for immuno-intensity quantification. Intensity of stain was measured using reference shades of nucleus (hematoxylin) and protein of interest (DAB/Fast red). Immune infiltrates and stromal cells were excluded from the analysis by setting a threshold on the intensity of hematoxylin, nuclei size, ferret ratio and eccentricity of the cells. Mean intensity per unit area of each stain was quantified by Histoquest software and was used for determining the difference in CXCR6, CXCL16 and ADAM10 expression.

### Reverse transcription–PCR analysis

Total RNA from cultured cells was isolated using the Tri reagent (Sigma, St. Louis, MO) and was resuspended in RNA Secure (Ambion). Genomic DNA contamination was removed from the samples by treatment with RNase-free DNase (Invitrogen) for 30 minutes at 37 °C. Equal amounts of total cellular RNA (1 μg) was reverse transcribed using iScript cDNA synthesis kit (Bio-Rad, Hercules, CA) as per manufacturer’s protocol. Transcribed cDNAs were used for RT-qPCR using SYBR Green PCR Master Mix reagent (Bio-Rad, Hercules, CA) in a 25 µl reaction volume with specific primers for human MMP-1, MMP-2, MMP-3, MMP-8, MMP-9, MMP-10, MMP-11, MMP-13 and 18 S rRNA, respectively. Relative quantities of specific PCR product were determined using the 2^−ΔΔCT^ method.

### Flow cytometry analysis of CXCR6 and CXCL16 expression in normal ovarian epithelial cells and ovarian cancer cells

Phycoerytherin (PE) conjugated mouse anti-human CXCR6 antibody, Allophycocyanin (APC) conjugated rat anti-human CXCL16 and their isotype controls PE-conjugated IgG2b, APC-conjugated IgG2a were purchased from R&D Systems (Minneapolis, MN). Culture media from OvCa (SKOV-3 and OVCAR-3) and IOSE-7576 cells was removed and cells were washed with PBS and harvested using Accutase (Corning), subsequently washed with FACS buffer (2% FBS in PBS). 10^5^ cells were treated with Fc Block (PharMingen) for 15 minutes at RT. Then cells were stained with PE-conjugated mouse anti-human CXCR6 and APC-conjugated rat anti-human CXCL16 on ice for 40 minutes. Controls were stained with PE and APC-conjugated isotype controls. After staining unbound antibody was washed with 500 μl FACS buffer and resuspended in 500 μl of 1X fixation buffer (Invitrogen, Frederick, MD) on ice for 10 min. Subsequently, cells were washed and resuspended in FACS buffer. Flow cytometry analysis of CXCR6 and CXCL16 was performed by acquiring 10,000 events using Guava EasyCyte (millipore). Analysis was done using FlowJo software.

### ELISA assay for soluble CXCL16 and MMPs

Normal ovarian and OvCa cells (SKOV-3 and OVCAR-3) were seeded in 1 ml of respective culture medium containing 2% FBS in six well culture plate (10^5^ cell/well). Conditioned media was collected after 24 h culture. Concentration of CXCL16 in the conditioned media was quantified using CXCL16 ELISA (R&D Systems, USA) as per manufacturer’s instruction. Levels of MMPs produced by the OvCa cells in response to CXCL16 were quantified in culture media. Briefly, SKOV-3 and OVCAR-3 cells were cultured in tissue culture flask in their respective culture media (10^6^ cell/T25 flask). Once cell reached at 70% confluency CXCL16 (100 ng/ml) was added to the media (3 ml) for 24 hours, media from untreated cells were used as control. ELISA assay was used to quantify the MMP produced with or without CXCL16 treatment in conditioned media using Quantikine DuoSet ELISA Kit (R&D Systems, USA) as per manufacturer’s instruction.

### Zymograms

SDS-PAGE gels (1.5 mm in thickness) containing 0.2% (wt/vol) gelatin or 0.1% (wt/vol) casein or 1 mg/ml collagen were prepared. 50 µg of conditioned media protein was loaded with equal volume of SDS-PAGE sample buffer without 2-mercaptoethanol for ~30 min at 37 °C. After electrophoresis, gels were washed in 2.5% (vol/vol) Triton X-100 for 30 min and then incubate in the incubation buffer (Biorad)  at 37 °C overnight for under shaking. After incubation, gels were stained with Coomassie Brilliant Blue R and followed by destaining.

### Tumor cell migration and invasion assays

Cell migration and Matrigel invasion chambers were obtained from Corning. Warm (37 °C) serum free bicarbonate based medium (DMEM) was added to the transwells for 2 hours in humidified tissue culture incubator, 37 °C, 5% CO_2_ atmosphere to hydrate. After hydration, media was gently aspirated from bottom and top chambers. 800 ul of respective medium containing 2% FBS supplemented with or without CXCL16 (100 ng/ml) was added to the bottom chamber. 10^4^ cancer cells treated with or without mouse anti-human CXCR6 antibodies (1 µg/ml) in 400 μl 2% FBS containing media were added to the top chamber of the inserts and incubated overnight. Thereafter, the cells from the top chamber were removed using cotton-tipped swab and the cells that migrated or invaded through and to the bottom surface of the inserts were fixed with 100% methanol for 2 minutes, stained for 30 minutes in 1% crystal violet and rinsed twice with distilled water. Migrated and invaded cells were then counted microscopically from different fields. These assays were repeated three times.

### Statistical analyses

Unpaired non-parametric Mann Whitney U test was used to compare CXCR6, N terminal CXCL16, C terminal CXCL16 and ADAM10 immunostaining in OvCa tissues of different histology types using GraphPad Prism 6 software. Student’s t test was used to determine statistical significance for expression data, migration, invasion and ELISA. Results were declared significant when p-value < 0.05. All *in vitro* experiments were repeated three times.

## Supplementary information

Supplementary figure 1

## Data Availability

We would be happy to provide any additional details not available in this manuscript.
