# Supplementary figures and images for: Higher CXCL16 exodomain is associated with aggressive ovarian cancer and promotes the disease by CXCR6 activation and MMP modulation
Source: Sci Rep. 2019 Feb 21;9:2527. doi: 10.1038/s41598-019-38766-6 (PMC6385302; doi:10.1038/s41598-019-38766-6)

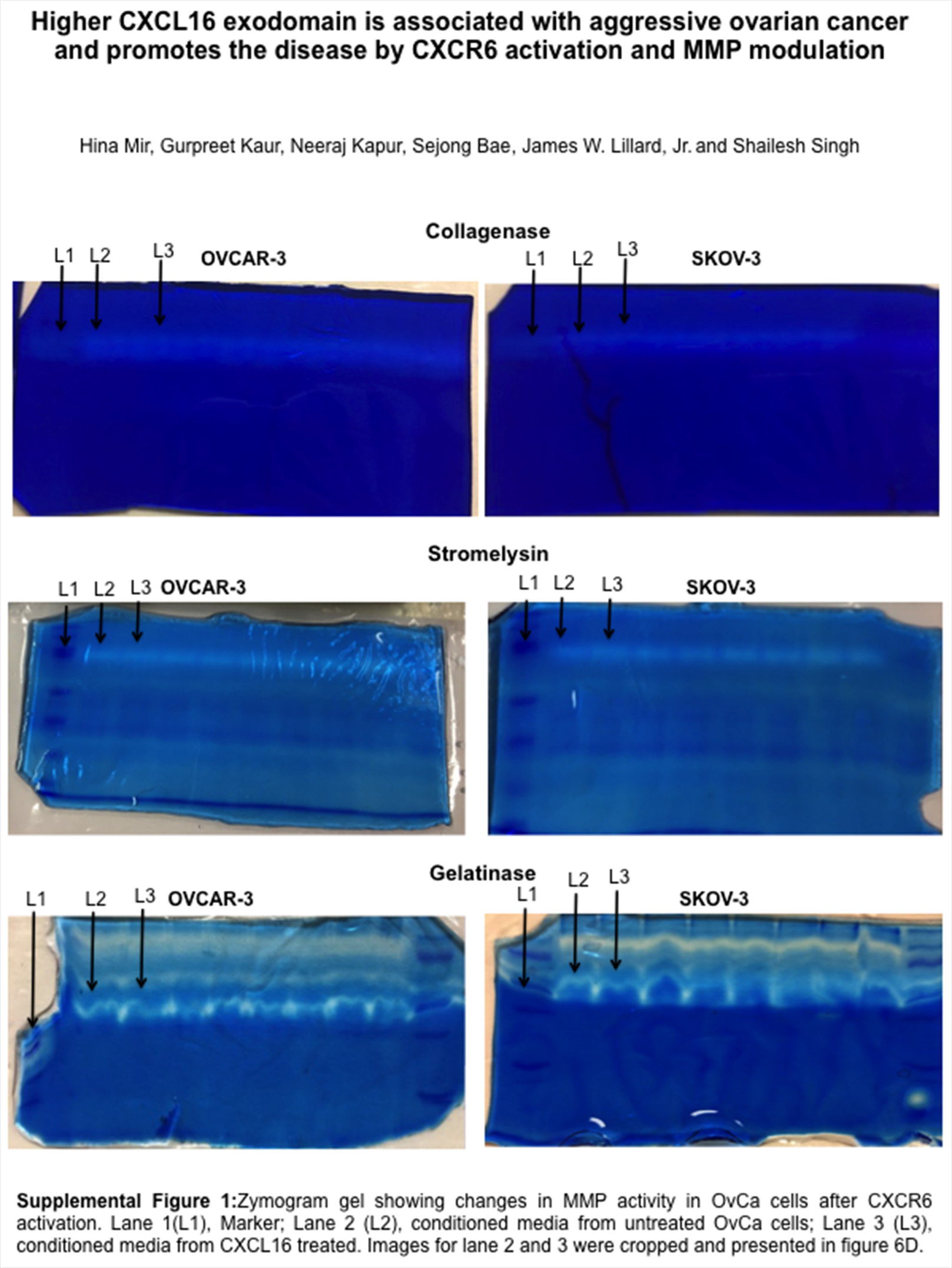

Supplement: Supplementary file 1 — Supplementary figure 1 [file 41598_2019_38766_MOESM1_ESM.tif]
